# Clinical Outcomes of Aberration-Free All Surface Laser Ablation (ASLA) vs. Aberration-Free ASLA Assisted by Smart Pulse Technology in High Myopia: A One-Year Follow-Up Study

**DOI:** 10.1155/2021/2588765

**Published:** 2021-10-18

**Authors:** XiaoHao Du, Jia Zhang, Meng Su, WenJia Cao, Shuang Zeng, QinMei Wang, Ioannis M. Aslanides, ShiHao Chen

**Affiliations:** ^1^School of Ophthalmology and Optometry, Wenzhou Medical University, Wenzhou, Zhejiang, China; ^2^Henan Provincial People's Hospital, Henan Provincial Eye Hospital, Zhengzhou, Henan, China; ^3^Eye Hospital of Wenzhou Medical University, Wenzhou, Zhejiang, China; ^4^Emmetropia Mediterranean Eye Institute, Heraklion, Crete, Greece; ^5^Taizhou Eye Hospital, Taizhou, Zhejiang, China

## Abstract

**Purpose:**

To compare the clinical outcomes of aberration-free all surface laser ablation (ASLA) with and without the use of smart pulse technology (SPT) in high myopia.

**Methods:**

This study retrospectively analyzed 138 eyes (138 patients, only the right eye was selected) treated for high myopia (spherical equivalent ≥−6.00 diopters) using aberration-free ASLA (non-SPT group; 85 eyes) and aberration-free ASLA assisted by SPT (SPT group; 53 eyes). Examinations such as visual acuity, refraction, and haze were performed before the 12-month follow-up. Corneal epithelial healing time was assessed in the first postoperative day. Visual acuity and refraction examination were performed at 7 days and 1, 3, 6, and 12 months postoperatively. Corneal haze was evaluated in 1, 3, 6, and 12 months. Safety, efficacy, and corneal wavefront aberrations were assessed 12 months after the treatment.

**Results:**

At 12 months postoperatively, 60% versus 40% of eyes achieved 20/16 Snellen lines or better, and 92% versus 82% of eyes achieved 20/20 Snellen lines or better visual acuity in the SPT and the non-SPT groups, respectively. The average postoperative epithelial healing time was 3.75 ± 1.00 days in the SPT group and 3.73 ± 1.30 days in the non-SPT group (*P* ≥ 0.05). The safety and the efficacy index of the SPT group were better than those of the non-SPT group in the follow-ups. The attempted spherical equivalent before the surgery and the achieved spherical equivalent at 12 months were comparable between the two groups. Regarding the aberrations, the results of Coma 90° in the SPT group were better than those in the non-SPT group (*P* ≤ 0.05), but the increase of RMS HOAs (root mean square higher order aberrations), Coma 0°, and spherical aberration postoperatively had no statistical difference between the two groups (*P* ≥ 0.05). *Conclusions:* Both aberration-free ASLA with and without SPT showed favorable safety, effectiveness, and predictability within 12 months for high myopia. And, ASLA using SPT might have potential advantages in the long-term visual quality.

## 1. Introduction

The term transepithelial photorefractive keratectomy (TransPRK) was firstly introduced by Alio in 1993s [1]. Differing from the chemical or mechanical methods used in earlier conventional surface surgery, TransPRK was a two-step process [2], including epithelial removal followed by stromal ablation. Subsequently, a series of improvements were followed such as one-step TransPRK, reverse one-step TransPRK, and smart pulse technology-assisted TransPRK [3–7]. Due to the quick vision recovery, the latest TransPRK approach has gradually been widely recommended by clinicians [5, 8].

In 2014, a new laser ablating mode using a fullerene three-dimensional model, referred to as smart pulse technology (SPT), was introduced. It is available on the SCHWIND Amaris platform (SCHWIND eye-tech-solutions GmbH, Kleinostheim, Germany) termed transepithelial all surface laser ablation (ASLA) and replaced the original method of TransPRK based on a grid-like corneal representation mode by software update. The advantages of SPT-assisted ASLA (SPT-ASLA) such as early postoperative vision recovery, pain and epithelial healing, corneal asphericity, and reduction of the irregularity of the stromal surface have been reported in some studies [6, 9, 10]. The results of the available research indicate that SPT-ASLA might optimize the geometric arrangement of pulses during laser ablation to make the corneal bed smoother, leading to earlier postoperative visual recovery, faster corneal re-epithelialization, and less haze occurrence than surface ablation without the SPT. However, there are few studies on the difference between SPT-ASLA and non-SPT-ASLA in the correction effect of high myopia. In this study, we aim to compare the results of those two ablation modes in the correction of high myopia.

The aim of this study was to comparatively evaluate the long-term clinical outcomes of aberration-free SPT-ASLA versus non-SPT-ASLA in patients with high myopia.

## 2. Materials and Methods

### 2.1. Patients

This comparative observational case series study was to retrospectively analyze 138 eyes (138 patients, only the right eye was selected) which were included in two groups of SPT-ASLA (SPT group; 53 eyes) and non-SPT-ASLA (non-SPT group; 85 eyes) and compare their long-term efficacy and safety. The cohorts of both groups comprised patients with high myopia who underwent SPT-ASLA or non-SPT-ASLA treatment at the department of refractive surgery of Eye Hospital, WMU, from 2013 to 2016 and met the screening criteria. The inclusion criteria included spherical equivalent of more than −6.00 diopters (D), age more than 18 years, stable refraction for at least 1 year, and the washout period for soft contact lens, hard contact lens, and orthokeratology was more than 2 weeks, 4 weeks, and 12 weeks, respectively. The exclusion criteria included keratoconus or abnormal corneal topography, previous history of intraocular or corneal surgery, ocular infection or active inflammation, autoimmune diseases, and pregnant or nursing women.

This study was reviewed and approved by the ethics committee of the Eye Hospital, WMU (Ethical approval number: 2019-197-k-177). Each patient was informed about the purpose and the procedure of this study, and informed consent was obtained before surgery.

### 2.2. Preoperative and Postoperative Assessment

All patients received detailed ophthalmological examination before surgery including uncorrected distance visual acuity (UDVA), corrected distance visual acuity (CDVA), refraction (manifest and cycloplegic refraction were performed by an optometrist), autorefractometry (RT-5100, NIDEK, Japan) such as sphere (Sph), cylinder (Cyl), and spherical equivalent (SE), slit lamp examination (SL-2G; TOPCON, Japan), and Scheimpflug-based corneal topography (Pentacam HR; Oculus Optikgeräte GmbH, Wetzlar, Germany). Corneal wavefront aberrations were also measured using Pentacam.

All patients of the study were followed up for 7 days and 1, 3, 6, and 12 months postoperatively. The parameters included the corneal epithelial healing time, visual acuity, refraction, safety, efficacy, corneal haze (Fantes scale) [11], and corneal wavefront aberrations.

### 2.3. Surgical Procedure

Before surgery, each patient received oral vitamin C tablets twice a day for one week, artificial tears four times a day for three days, and 0.5% levofloxacin four times a day for two days. Prednisone tablets (30 mg) were taken on the morning of the day before surgery, the day of surgery, and the day after surgery.

Regarding the surgical procedure itself, after conventional disinfection of the conjunctiva and the eyelids, an eye speculum was used to expose the surgical area and alcaine eye drops were administered for surface anesthesia. Aberration-free treatment was performed by the AMARIS 750S excimer laser platform (SCHWIND eye-tech-solutions GmbH, Kleinostheim, Germany) with a single continuous profile. Both groups were treated using aberration-free profiles. In the first group, the SPT update was not installed yet (non-SPT-ASLA), while in the second one, the treatments were performed with the SPT installed (SPT-ASLA). After the laser ablation, 0.02% mitomycin-C was applied to infiltrate the substrate bed, and the eye was then irrigated with cold balanced salt solution. 0.5% tobramycin dexamethasone was applied on the stromal bed, and a bandage contact lens (Air Optix Night&Day Aqua Soft Contact Lenses; Alcon Laboratories, America) was applied on the cornea until the epithelial healing was complete.

After surgery, 0.5% tobramycin dexamethasone eye drops were applied every 2 hours on the day of the surgery and then four times a day for 1 week. The dosage was adjusted appropriately according to the corneal condition. 1 month postoperatively, it was changed to 0.1% fluorometholone eye drops three times a day and then gradually decreased to 1 drop/month until drug withdrawal for about 4 months after surgery. Artificial tears were applied once an hour for 3 days after the operation and then changed to four times a day for 6 months. Oral vitamin C tablets were taken 500 mg twice a day for two weeks postoperatively.

### 2.4. Statistical Analysis

The values are expressed as mean ± standard deviation (SD). The differences between preoperative and postoperative outcomes at 12 months were calculated using the paired Student's *t*-test or the Wilcoxon test, as needed. The group *t*-test or the Mann–Whitney *U*-test were used to compare the differences between the two groups, as needed. Repeated measures analysis of variance was applied to the data of multiple measurements. A *P* value of 0.05 or less was considered statistically significant. All analyses were performed by SPSS software (version 23; IBM Corporation, Armonk, NY).

## 3. Results

This study includes 53 eyes in the SPT group and 85 eyes in the non-SPT group. The preoperative baseline of the two groups is shown in Table 1.

### 3.1. Healing Time of Corneal Epithelium

**Figure 1:**
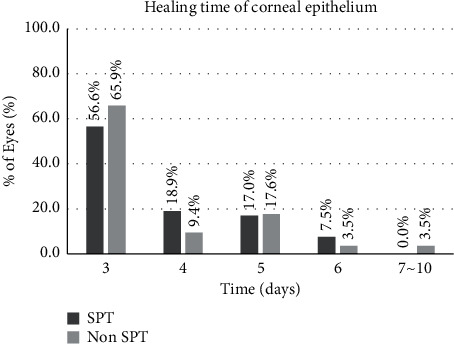
Postoperative healing time of corneal epithelium in the SPT and the non-SPT groups. SPT = smart pulse technology.

The mean postoperative healing time of the corneal epithelium was 3.75 ± 1.00 days and 3.73 ± 1.30 days in the SPT and the non-SPT group, respectively (*P*=0.399). Epithelial healing was observed at 3 days after treatment in most eyes of both groups. The healing process was completed in 3 to 6 days after surgery in the SPT group and in up to 10 days in the non-SPT group (Figure 1).

### 3.2. Visual Acuity and Refraction

At 12 months after the treatment, the change of UDVA, sphere, cylinder, and spherical equivalent has no statistical difference between two groups. The improvement of CDVA in SPT group seems statistically better than that in the non-SPT group (Table 2).

### 3.3. Safety and Efficacy

**Figure 2:**
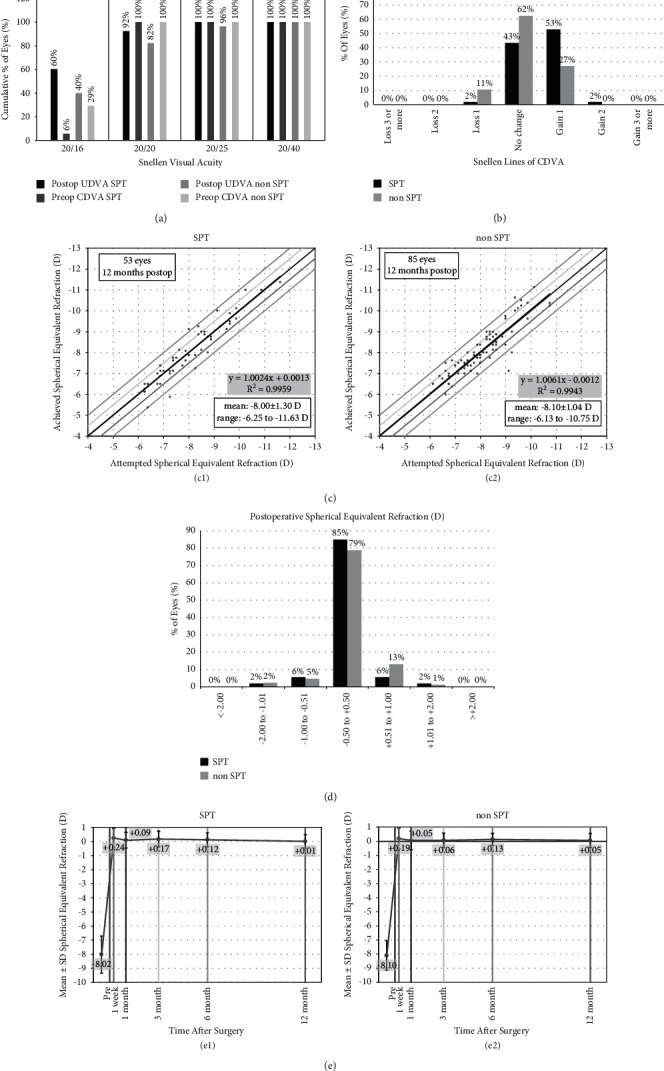
(a) Efficacy of all surface laser ablation (ASLA) assisted by smart pulse technology (SPT group) versus ASLA without SPT (non-STP group) 12 months postoperatively. (b) Change in corrected distance visual acuity. (c) Attempted vs. achieved spherical equivalent (SE) refraction in the SPT group and the non-SPT group. (d) SE refraction accuracy. (e) Preoperative, 1 week, and 1, 3, 6, and 12 months SE values in the SPT group and the non-SPT group.

The distribution diagram of UDVA in 12 months postoperative is shown in Figure 2(a). There were 60% of eyes and 40% of eyes which achieved 20/16 (Snellen lines) or better in the SPT group and in the non-SPT group, respectively. In addition, there were 92% and 82% of the eyes which achieved 20/20 (Snellen lines) or better in the SPT and non-SPT group, respectively. Regarding CDVA 12 months postoperatively, 2% of eyes in the SPT group and 11% of the eyes in the non-SPT group had lost up to 1 Snellen lines, but there were no eyes with more than 2 lines lost (Figure 2(b)). The safety index (postoperative mean CDVA/preoperative mean CDVA) and the efficacy index (postoperative mean UDVA/preoperative mean CDVA) of the SPT group were statistically better than those of the non-SPT group (Table 3).

### 3.4. Accuracy and Stability

Attempted spherical equivalent before surgery was −8.00 ± 1.30 diopter (D) in the SPT group, which was not different compared to the one in the non-SPT group (−8.10 ± 1.04 D, *P*=0.456). The achieved spherical equivalent at 12 months after the surgery was −8.01 ± 1.41 D in the SPT group, which was not statistically different to the one in the non-SPT group (−8.15 ± 1.15 D, *P*=0.493). The predictive curve of refraction of the two groups had similar linear relationships ((c1) and (c2) in Figure 2(c)). 12 months postoperatively, 85% of the eyes in the SPT group were within 0.50 D of emmetropia, whereas in the non-STP group, 79% of the eyes were within 0.50 D of emmetropia (*P*=0.107) (Figure 2(d)).

The refractive stability of both groups (SPT and non-SPT) is shown in (e1) and (e2) in Figure 2(e), respectively.

### 3.5. Haze

**Figure 3:**
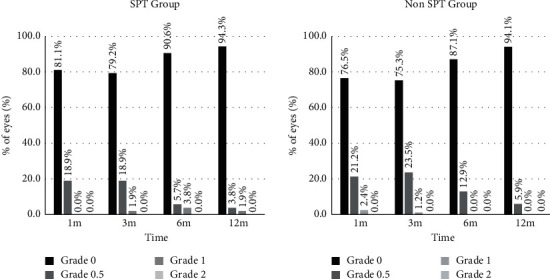
Variable occurrence rate of haze after SPT or non-SPT. SPT = smart pulse technology.

The occurrence rate of haze is shown in Figure 3. The haze score was similar in both groups (*P* > 0.05). More than 20% of eyes developed mild haze in both groups at 1 month and 3 months after surgery, and most of them were 0.5 grade (haze traces) and were decreased gradually during the follow-up of the next month.

### 3.6. Corneal Aberrations

The post-12-month root mean square higher order aberrations (RMS HOA) in the two groups were statistically increased than those in the preoperative stage (*P* < 0.001) (Table 4). The values of spherical aberration and the horizontal coma (Coma 0°) in the two groups were increased postoperatively (*P* < 0.001), but there was no statistical difference between the SPT group and the non-SPT group. The values of the postoperative vertical coma (Coma 90°) in the two groups were statistically increased than preoperatively (*P* < 0.05). Nevertheless, the increase was smaller in the SPT group (*P*=0.003).

## 4. Discussion

Safety, efficacy, and predictability of TransPRK have been proved by several previous studies [12–16]. The application of smart pulse technology (SPT) in all surface laser ablation (ASLA) causes better early postoperative visual recovery and faster epithelial healing [6]. This study mainly compared the long-term results of aberration-free ASLA with and without the use of SPT for high myopia to make sure which might have better outcomes in high myopia.

Serrao and Lombardo [17] showed that the more regular the surface of the corneal stroma after PRK was, the faster the healing of the corneal epithelial would be. Meanwhile, the postoperative healing of the epithelium in eyes with high myopia was significantly slower than in cases with low myopia. SPT uses three-dimensional model of fullerene to optimize the geometric arrangement of pulses during laser ablation, which theoretically provides a smoother corneal stromal bed and faster healing of the corneal epithelium. Another study by Aslanides [6] showed the outcomes of 89 eyes (49 eyes in the SPT group, mean spherical equivalent: −3.87 ± 2.21D; 40 eyes in the non-SPT group, mean spherical equivalent: −4.8 ± 1.86D). Their results of the epithelial healing time showed that all of the eyes were healed within 3 days in both groups, and it was better in the SPT group than in the non-SPT group. However, in our study, there was no statistically significant difference in mean epithelial healing time between the two groups, and the healing process took longer. The reason might be that the limitation of our study was patients with high myopia (mean spherical equivalent ＞−6.00 D) and a relatively long time was spent for corneal epithelial healing and remodeling, which might weaken the effects of the SPT in making the ablation surface smoother and easier to heal the epithelium. Meanwhile, the range of treatment areas was also a factor affecting the results of the study.

Almost all the results of the safety index and the efficacy index were also superior in the SPT group. Previous studies have shown that the smoothness of regenerated epithelium strongly depends on the roughness of the residual corneal stromal surface [18–21]. If the corneal epithelial cells regenerated and migrated in a smoother plane, the connections between the cells are also more regular. In addition, the fibroblast proliferation and corneal opacity would be reduced, which may improve the visual quality to some extent and thus affect the postoperative eyesight. This fact could explain the better results in the SPT group.

As stated in previous studies, surgical accuracy becomes more elusive as the degree of correction increases. [21, 22] Meanwhile, the deeper the ablation, the more the hyperplasia of the epithelium, and the hyperplasia of corneal epithelium is associated with refractive regression [23]. This may indicate that the prediction of long-term postoperative refractive results of high myopia is more difficult than that of low to moderate myopia. In our study, the predictive curves drawn by the follow-up results of the two groups after 1 year postoperatively were similar. More than 90% of eyes were within 1.0 D at the 1-year follow-up which may provide an encouraging basis for the application of ASLA to patients with high myopia.

The proliferation of fibroblast and corneal turbidity would be reduced due to a smooth stromal surface [24–27]; as a consequence, haze in the SPT group theoretically was smaller than that in the non-SPT one. In Aslanides' and Vinciguerra's study [6, 10], there was no statistically difference between the SPT group and non-SPT group, which was similar to our results. Haze occurred in more than 20% of eyes in both groups at 1 month and 3 months after surgery which may be seem larger than the occurrence in previous research, but most of them were 0.5 level that barely affected vision and were gradually decreased and disappeared after the use of 0.1% fluorometholone eye drops. We consider that the main reason for this result is that haze is more likely to occur after correction of high myopia [26–28]. There were other objective factors which may also affect the results, such as the slight difference of clinician judgment for haze's corneal grade and patient's compliance.

Previous studies have reported that an increase in RMS HOAs of the anterior cornea was linearly correlated with the preoperative spherical equivalent and the central corneal ablation depth according to these results [29–32]. Juhasz et al. [30] found that RMS HOA values showed a 2.4-fold growth when the ablation depth is ＞77 um. In our study, RMS HOAs in both groups were also significantly increased about 2.5∼3 times postoperatively, and the ablation depth was about 160∼170 *μ*m. The diopter of myopia and the ablation depth should be considered as important factors for increased HOAs. We expected that the introduction of SPT could reduce the postoperative spherical aberration by reducing the roughness of the cutting surface, but SPT did not seem to make an impact on the results in our study.

**Figure 4:**
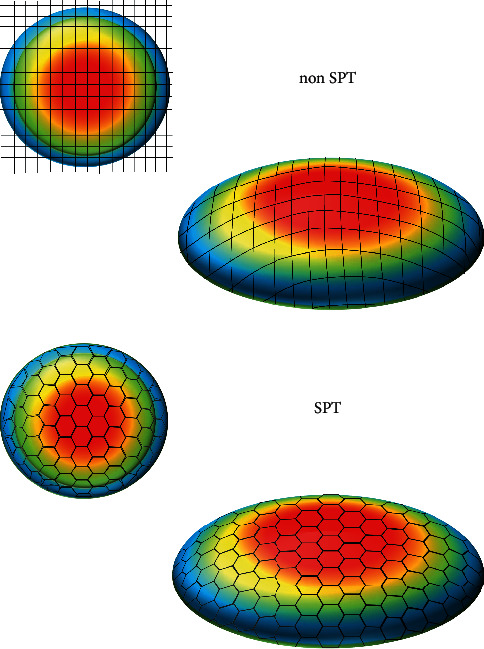
In the mode of non-SPT, the closer to the corneal periphery area is, the greater the energy loss is. While in the SPT mode, the energy loss is relatively reduced. SPT = smart pulse technology.

The results of horizontal coma (coma 0°) had significant difference between preoperation and postoperation both in the SPT and non-SPT groups, nor was there any significant difference between the two groups. However, the postoperative results of vertical coma (coma 90°) in both groups were significantly larger than the preoperative results, and the increase of coma 90°in the SPT group was significantly less than that in non-SPT group. We have several theories about this outcome. On the one hand, higher order aberration effects (spherical aberration and coma) are mainly derived from the edge effect, that is, the change of local curvature from the optical region to the transition region and from the transition region to the untreated corneal region [33]. In normal mode, when the laser ablation is near the periphery of the cornea, the ablating energy per unit area is lost due to the increase of the spot area [34], a phenomenon known as “the cosine effect” (Figure 4). In addition, the difference in the radius of local curvature between the upper and lower corneal areas leads to the difference in the energy loss of the surrounding area between the upper and lower corresponding region. As a result, the vertical coma would be inducted by the difference of local corneal ablation between the upper and lower parts. However, the improvement by SPT on the pattern and arrangement of laser spot could reduce the loss of laser energy which may make the energy per ablating area more uniform. In this mode, the ablating inhomogeneity caused by the difference of corneal curvature between the upper and lower areas may be relatively reduced, so that the increase of the asymmetry between the upper and lower parts may be reduced and the induction of vertical coma may be relatively reduced vs. non-SPT. On the other hand, since the distribution of corneal epithelium is asymmetric, the upward side is thick and the downward side is thin, and the difference in corneal remodeling process between the upper and lower areas may introduce more asymmetry and lead to the increase of coma 90°. However, SPT can make epithelial healing and remodeling more rapid and uniform by reducing the roughness of the corneal stromal surface, which may also reduce the additional coma introduced by the difference in thickness of the upper and lower corneal epithelium requiring remodeling.

More patients and longer follow-up are the next focus of this study.

## 5. Conclusions

Both SPT and non-SPT aberration-free ASLA techniques showed favorable safety, effectiveness, and predictability within 12 months for high myopia. ASLA using SPT might have potential advantages in the long-term visual quality.

## Figures and Tables

**Table 1:** Baseline preoperative values^a^.

Baseline preoperative values	SPT group	Non-SPT group	*P*
Number of eyes	53	85	
Mean age (years)	26.25 ± 5.64	24.62 ± 4.91	0.082
Mean preoperative logMAR UDVA	1.294 ± 0.245	1.280 ± 0.269	0.725
Mean preoperative logMAR CDVA	−0.007 ± 0.017	−0.024 ± 0.034	0.02^b^
Mean preoperative refractive errors (D)			
Sphere	−7.60 ± 1.31	−7.60 ± 1.06	0.925
Cylinder	−0.83 ± 0.64	−1.00 ± 0.71	0.206
Spherical equivalent	−8.02 ± 1.32	−8.10 ± 1.05	0.507
Optical zone diameter (mm)	6.12 ± 0.26	6.18 ± 0.26	0.494
Treatment diameter (mm)	7.91 ± 0.33	8.09 ± 0.33	0.001^b^
Ablation depth (*μ*m)	160.85 ± 13.05	171.49 ± 11.25	＜0.001^b^
Residual stromal bed thickness (*μ*m)	345.83 ± 21.44	330.11 ± 18.71	＜0.001^b^

SPT = smart pulse technology; UDVA = uncorrected distance visual acuity; CDVA = corrected distance visual acuity. ^a^Values are presented as mean ± standard deviation. ^b^Significant difference between the SPT and non-SPT groups (Student's *t*-test/Mann–Whitney *U*-test).

**Table 2:** Visual acuity and refraction outcomes^a^.

Parameter	Group	Preoperative	12 months	*P*
LogMAR UDVA	SPT	1.294 ± 0.245	−0.040 ± 0.054	0.533
	Non-SPT	1.280 ± 0.269	−0.009 ± 0.080	

LogMAR CDVA	SPT	−0.007 ± 0.017	−0.046 ± 0.045	0.004^b^
	Non-SPT	−0.024 ± 0.034	−0.038 ± 0.041	

Sphere	SPT	−7.60 ± 1.31	0.24 ± 0.50	0.770
	Non-SPT	−7.60 ± 1.06	0.26 ± 0.51	

Cylinder	SPT	−0.83 ± 0.64	−0.45 ± 0.34	0.169
	Non-SPT	−1.00 ± 0.71	−0.42 ± 0.28	

Spherical equivalent	SPT	−8.02 ± 1.32	0.01 ± 0.47	0.493
	Non-SPT	−8.10 ± 1.05	0.05 ± 0.50	

SPT = smart pulse technology; UDVA = uncorrected distance visual acuity; CDVA = corrected distance visual acuity. ^a^Values are presented as mean ± standard deviation. ^b^Significant difference between the SPT and non-SPT groups (Student's *t*-test/Mann–Whitney *U*-test).

**Table 3:** Changes of safety and efficacy index after 12 months in SPT group and non-SPT group^a^.

Time	Safety index			Efficacy index		
	SPT group	Non-SPT group	*P*	SPT group	Non-SPT group	*P*
7 days	0.81 ± 0.17	0.71 ± 0.17	＜0.001^b^	0.79 ± 0.22	0.65 ± 0.17	0.001^b^
1 month	0.99 ± 0.08	0.91 ± 0.14	＜0.001^b^	0.98 ± 0.13	0.84 ± 0.24	＜0.001^b^
3 months	1.07 ± 0.11	1.02 ± 0.13	0.033^b^	1.09 ± 0.18	0.99 ± 0.19	0.005^b^
6 months	1.08 ± 0.11	1.07 ± 0.12	0.477	1.08 ± 0.15	0.99 ± 0.18	0.009^b^
12 months	1.10 ± 0.12	1.04 ± 0.11	0.004^b^	1.09 ± 0.14	0.98 ± 0.17	＜0.001^b^

SPT = smart pulse technology. Safety index = postoperative mean CDVA/preoperative mean CDVA. Efficacy index = postoperative mean UDVA/preoperative mean CDVA. ^a^Values are presented as mean ± standard deviation. ^b^Significant difference between the SPT and non-SPT groups (Student's *t*-test/Mann–Whitney *U*-test).

**Table 4:** Anterior corneal aberration values^a^.

Parameter	Group	Preoperative	After 12 months	*P*	△(12-month postoperative vs. preoperative)
RMS HOA	SPT group	0.35 ± 0.09	0.89 ± 0.24	＜0.001^c^	0.54 ± 0.25
	Non-SPT group	0.38 ± 0.10	1.03 ± 0.36	＜0.001^c^	0.65 ± 0.39
	*P* ^*∗*^	0.069	0.059		0.238

Spherical aberration	SPT group	0.25 ± 0.09	0.73 ± 0.19	＜0.001^c^	0.48 ± 0.20
	Non-SPT group	0.22 ± 0.08	0.77 ± 0.21	＜0.001^c^	0.56 ± 0.22
	*P* ^*∗*^	0.024^b^	0.461		0.091

Coma 0°	SPT group	−0.05 ± 0.10	−0.29 ± 0.26	＜0.001^c^	−0.24 ± 0.28
	Non-SPT group	−0.11 ± 0.10	−0.31 ± 0.39	＜0.001^c^	−0.21 ± 0.39
	*P* ^*∗*^	0.001^b^	0.840		0.231

Coma 90°	SPT group	−0.00 ± 0.15	−0.15 ± 0.42	0.012^c^	−0.15 ± 0.39
	Non-SPT group	−0.04 ± 0.20	−0.40 ± 0.51	＜0.001^c^	−0.36 ± 0.47
	*P* ^*∗*^	0.234	0.001^b^		0.003^b^

RMS HOA = root mean square higher order aberrations; SPT = smart pulse technology. ^*∗*^Compared with the SPT group and non-SPT group. ^a^Values are presented as mean ± standard deviation. ^b^Significant difference between the SPT and non-SPT groups (Student's *t*-test/Mann–Whitney *U*-test). ^c^Significant difference between preoperative and postoperative (Student's *t*-test/Wilcoxon test).

## Data Availability

The data are available from the corresponding author upon reasonable request.
